# Continuing Professional Development – Medical Imaging

**DOI:** 10.1002/jmrs.894

**Published:** 2025-06-11

Maximise your continuing professional development (CPD) by reading the following selected article and answering the five questions. Please remember to self‐claim your CPD and retain your supporting evidence. Answers will be available via the QR code and published in JMRS—Volume 72, Issue 4, December 2025.

## Radiographer Preferences for Shoulder X‐Ray Imaging in Australia: A National Survey

Zoe Moran, Amber Loh, Annie K. Lewis, Paul Kelly, Amy M. Dennett, https://doi.org/10.1002/jmrs.881.

1. Which organisation developed the published guidelines that inform the gold standard protocol for shoulder imaging?
  - European Society of Radiology
  - Australasian Radiographers Association
  - North American Alliance of Radiographers for the Shoulder
  - Guidelines have not yet been published
2. According to this study, which of the following statements is correct?
  - Radiographers were consistent in their approach to imaging the shoulder.
  - Radiographers were consistent in their approach for arthritis but not for trauma.
  - Radiographers consistently rely on published guidelines when selecting imaging views.
  - Radiographers showed variability in the views chosen to image common shoulder pathologies.
3. What was the average number of imaging views selected by participants for shoulder pathology in this study?
  - 1
  - Between 2 and 4
  - Between 6 and 8
  - 7
4. In this study, participants most commonly selected modified imaging views for which shoulder condition?
  - Arthritis
  - Impingement
  - Shoulder replacement
  - Glenohumeral dislocation
5. Based on the findings of this study, which standard radiographic view was most commonly selected for patients with general shoulder trauma?
  - Outlet/Neers view
  - AP general survey
  - True AP/Grashey (neutral rotation)
  - Lateral Y scapular view

## Answers

Scan this QR code to find the answers.

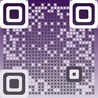
